# Influence of maxillary posterior dentoalveolar discrepancy on angulation of maxillary molars in individuals with skeletal open bite

**DOI:** 10.1186/s40510-016-0147-8

**Published:** 2016-11-01

**Authors:** Luis Ernesto Arriola-Guillén, Aron Aliaga-Del Castillo, Carlos Flores-Mir

**Affiliations:** 1 Division of Orthodontics, Faculty of Dentistry, Universidad Científica del Sur - UCSUR, Calle Los Girasoles # 194, Dpto. # 302, Urb. Residencial Los Ingenieros de Valle Hermoso, Santiago de Surco, Lima Perú; 2 Department of Orthodontics, Bauru Dental School, University of São Paulo, Bauru, Brazil; 3 Faculty of Medicine and Dentistry, University of Alberta, Edmonton, Alberta Canada

**Keywords:** Posterior discrepancy, Molar angulation, Open bite, Cephalometry

## Abstract

**Background:**

The aim of this study was to determine the effect of the maxillary posterior dentoalveolar discrepancy (MPDD) on the angulation of maxillary molars in open bite subjects.

**Methods:**

Pre-treatment lateral cephalograms of 90 young adults with skeletal open bite were examined. The sample initially included six groups categorized according to MPDD condition (present or absent) and sagittal skeletal facial growth patterns (classes I, II, or III). Then, the sample was separated into two groups according to MPDD (present = 50, absent = 40). When the eruption of the maxillary third molar was apparently blocked by the presence of an erupted second molar, a MPDD was considered. Maxillary molar angulation was measured. Independent *T* test was performed to determine differences between the groups considering MPDD condition. Principal component analysis (PCA) and multivariate analysis (MANCOVA) test were also developed.

**Results:**

A decreased molar angulation was found in all groups with MPDD (overall *p* < 0.001, class I—*p* < 0.001, class II—*p* < 0.001, and class III—*p* < 0.05). The maxillary first and second molars angulations were lower between approximately 7° and 14° in cases with posterior discrepancy. The PCA was used to reduce the number of initial cephalometric variables; thereafter, a MANCOVA test was applied. Significance was only found for MPDD (*p* < 0.001), APDI (*p* = 0.001), and ratio (A′6′/A′P′) (*p* = 0.026) for maxillary first molar angulation and APDI (*p* = 0.011) and MPDD (*p* < 0.001) for maxillary second molar angulation.

**Conclusions:**

The MPDD generates a major mesial displacement of the second and first molar roots with a concurrent simultaneous distal angulation of the associated crowns in individuals with skeletal open bite.

## Background

The angulation of posterior molars has been studied in several papers [1–4] including individuals with different sagittal malocclusions associated with different sagittal and vertical growth patterns, but none so far has evaluated the specific impact of maxillary posterior dentoalveolar discrepancies (MPDD) (apparent lack of space for erupting third molars by inadequate pathway eruption) on molar angulations. It has been suggested that posterior discrepancies may be related to crowding relapse and third molar impaction [5–10]. Nevertheless, the majority of orthodontists and oral surgeons do not consider the preventive third molar extraction in order to prevent anterior crowding [11]. There are a few systematic reviews [12–14] in literature unsupportive on the role of the third molars in the development of late incisal crowding. In addition, it has been shown that a maxillary posterior discrepancy is not necessarily associated with increases in maxillary molar vertical eruption, overbite, or anterior lower facial height [15]. One hypothesis suggests that the posterior discrepancy should have an increase in the mesial angulation of the upper first and second molars (involving their crowns and roots) [6–10], while another hypothesis suggests that in MPDD cases, the pressure from the erupting maxillary third molar generates a mesial push over the second molar roots with a concurrent simultaneous distal tipping of their crowns [1].

Because of the existing controversy for either one of the described hypothesis, namely mesialization of posterior molars or a distoangulation of the molar crown with a concomitant mesialization of their roots, the purpose of this study was to determine the effect of the MPDD on the sagittal inclination of maxillary molars in open bite individuals with different sagittal malocclusions. If such associations existed, then this information could be useful for clinicians when treatment planning biomechanical approaches to cases with potential MPDD, especially in subject with skeletal open bite.

## Methods

This retrospective study was approved by the ethical committee of the School of Dentistry, Universidad Científica del Sur, Lima, Perú.

### Sample characteristics

The sample included 90 pre-treatment lateral cephalograms of Latin-American individuals (45 male, 45 female). These cases were part of a previously published [15] sample of cases. All the cephalograms were taken at maximum intercuspidation with the lips at rest in subjects aged 15 to 30 years old (21.50 ± 4.48). Imaging was performed with a digital cephalometric panoramic equipment (ProMax®, Planmeca, Finland) with settings set at 16 mA, 72 kV, and 9.9 s. Cephalometric analyses were performed digitally by two calibrated examiners with MicroDicom viewer software (version 0.8.1; Simeon Antonov Stoykov, Sofia, Bulgaria), without magnification, at a scale of 1:1.

Subjects with previous orthodontic treatment, tumors, infection or prosthetic molar reconstruction in the maxillary molar region and without maxillary third molars (extracted or missing) or any other missing/extracted permanent teeth were not considered.

Although a convenience sample of available records was used, sample size was calculated to demonstrate external validity. The sample size was calculated considering a mean difference of 10° in the maxillary second molar sagittal inclination as a clinically relevant difference between groups with and without MPDD. A standard deviation of 4° was considered (obtained from a preliminary pilot study) with a two-sided significance level of 0.01 and a power of 90 %. Although a minimum of five subjects per group was required, at least eight subjects per group were available. The calculated sample was 30 subjects; however, data from 90 subjects that met the selection criteria in a reference center of imaging were included.

### Sample grouping

The study sample included six groups categorized according to their MPDD condition (present or absent) and to their sagittal skeletal facial growth patterns (classes I, II, or III) [16–19] (Table 1).

**Table 1:** Sample distribution by group, sex, and age

Group	Male	Female	Total	Age^a^ Mean (SD)
OBCIG-PD	9	9	18	20.40 (4.67)
OBCIG-WPD	4	6	10	20.87 (4.79)
OBCIIG-PD	6	13	19	22.64 (5.39)
OBCIIG-WPD	11	11	22	21.74 (4.46)
OBCIIIG-PD	8	5	13	20.67 (3.83)
OBCIIIG-WPD	7	1	8	22.69 (3.79)
Total	45	45	90	

^a^Not significant based on independent *T* test according to posterior discrepancy by groups

*OBCIG* open bite class I group, *OBCIIG* open bite class II group, *OBCIIIG* open bite class III group, *PD* posterior discrepancy, *WPD* without posterior discrepancy

The definitions of the cephalometric points, distances, and angles [18–22] between them are shown in Table 2.

**Table 2:** Definitions of cephalometric points and angles used in this study

Angular measurements	Definition
SNA	The angle between points sella (S), nasion (N), and subnasal (A) in degrees [16]
SNB	The angle between points sella (S), nasion (N), and supra mental (B) in degrees [16]
ANB	The angle to assess the skeletal relationship between points A and B in degrees [16]
APDI	The anterior-posterior dysplasia indicator to assess the skeletal relationship and is obtained from the algebraic sum of the angles N-Pg-FH (facial plane) plus/minus the angle AB-facial lane (is positive when the point B is ahead of point A and is negative when the point A is ahead of point B) and plus/minus the angle FH-PP (palatal plane) (is negative when PP is tilted upward and positive when tilted down) [17]
FMP	The angle between the porion-orbital line and mandibular line in degrees
ODI	The overbite depth indicator to assess the tendency toward open bite is obtained from the algebraic sum of the angles AB-MP plus/minus the angle FH-PP (palatal plane) and is negative when PP is tilted upward and positive when tilted down [18]
Maxillary first molar angulation	The angle formed by the maxillary first molar axis (intercuspid groove-bifurcation) and the palatal plane (ANS-PNS), represented by a horizontal line
Maxillary second molar angulation	The angle formed by the maxillary second molar axis (intercuspid groove-bifurcation) and the palatal plane (ANS-PNS), represented by a horizontal line
Linear measurements	Definition
A′P′	The distance between the perpendicular extensions of points A and P on the palatal plane (A′P′) in millimeters: point A′ is the perpendicular projection of point A to the palatal plane and point P′ is the perpendicular projection of the posterior—most point of the maxillary tuberosity to the palatal plane [6, 9, 19]
A′6′	The distance between A′ and 6′ in millimeters, the anterior maxillary base length is defined by the measurement between A′ and 6′. Point 6′ is the perpendicular projection of the anterior-most point on the proximal surface of the maxillary first molar to the palatal plane [6, 9, 19]
Ratio (A′6′/A′P′)	The ratio of the anterior maxillary base length A′6′ to the maxillary base length A′P′ (A′6′/A′P′) [6, 9, 19]
Overbite	The overbite in millimeters is the distance between incisal edge of maxillary and mandibular central incisor, perpendicular to occlusal plane [20]
Lower anterior facial height (LAFH)	The length in millimeters of a line between points anterior nasal spine (ANS) and mental (Me) [21]
Ratio facial height (S-Go/N-Me × 100)	The ratio of posterior facial height and anterior facial height [22]

All subjects had a skeletal open bite (FMP angle greater than 26°, ODI lower than 72°, and lower anterior facial height greater than 67 mm) (Table 3).

**Table 3:** Sample characteristics by facial growth pattern and maxillary posterior dentoalveolar discrepancy

Measurement	Group	Mean	SD	Group	Mean	SD
SNA	OBCIG-PD	81.17*	2.89	OBCIG-WPD	84.54*	4.82
SNB		78.65	3.35		81.03	4.45
ANB		2.54	1.20		3.47	1.41
APDI		83.45	2.93		81.95	2.26
FMP		30.48	2.70		31.15	3.11
A′P′		42.45*	2.89		47.07*	3.10
A′6′		21.48	2.16		20.66	2.71
Ratio (A′6′/A′P′)		0.50*	0.04		0.44*	0.03
ODI		64.70	5.01		68.19	5.60
Overbite		−1.58	1.07		−2.50	1.52
Lower anterior facial height		67.99	4.88		69.31	3.85
Ratio facial height		60.73	3.04		62.50	3.22
SNA	OBCIIG-PD	80.97	3.09	OBCIIG-WPD	82.51	2.79
SNB		74.54	2.90		75.83	2.99
ANB		6.42	1.44		6.68	1.24
APDI		74.06	3.37		75.41	3.20
FMP		34.93	4.84		34.02	4.28
A′P′		43.62	3.63		45.96	2.72
A′6′		23.02*	2.68		21.23*	2.77
Ratio (A′6′/A′P′)		0.52*	0.04		0.46*	0.05
ODI		70.39	5.14		71.56	5.95
Overbite		−2.25	1.56		−2.99	1.29
Lower anterior facial height		70.54	5.69		73.44	5.12
Ratio facial height		58.50	3.41		59.70	3.23
SNA	OBCIIIG-PD	81.84*	3.27	OBCIIIG-WPD	78.22*	2.31
SNB		83.45*	4.16		80.14*	1.90
ANB		−1.61	1.36		−1.92	1.53
APDI		91.51*	4.48		87.67*	1.68
FMP		31.91	3.60		29.21	3.36
A′P′		43.88	5.00		44.06	4.32
A′6′		20.68	2.18		19.71	2.12
Ratio (A′6′/A′P′)		0.48*	0.06		0.44*	0.05
ODI		56.30*	4.55		62.99*	1.11
Overbite		−1.97	1.67		−1.78	1.54
Lower anterior facial height		70.37*	4.18		74.27*	2.55
Ratio facial height		59.84	2.36		60.52	1.65

*Significant based on Independent *T* test

Therefore, the groups were set as follows:

- ο Open bite class I group with maxillary posterior discrepancy OBCIG-PD (*n* = 18): ANB angle between 0° and 5°, antero posterior dysplasia indicator (APDI) of 81.4° ± 4°, angle class I malocclusion, bilateral class I molar relations, overjet between 1 to 5 mm, negative overbite greater than 0.5 mm, and diagnosed with maxillary posterior discrepancy
- ο Open bite class I group without maxillary posterior discrepancy (OBCIG-WPD) (*n* = 10): the same with the OBCIG-PD, but without posterior discrepancy
- ο Open bite class II group with maxillary posterior discrepancy (OBCIIG-PD) (*n* = 19): ANB > 5°, APDI < 75°, angle class II-1 malocclusion, bilateral class II molar relations, overjet greater than 5 mm, negative overbite greater than 0.5 mm, and diagnosed with maxillary posterior discrepancy
- ο Open bite class II group without maxillary posterior discrepancy (OBCIIG-WPD) (*n* = 22): the same with the OBCIIG-PD, but without posterior discrepancy
- ο Open bite class III group with maxillary posterior discrepancy (OBCIIIG-PD) (*n* = 13): ANB < 0°, APDI > 88°, angle class III malocclusion, bilateral class III molar relations, overjet lower than −1 mm, negative overbite greater than 0.5 mm, and diagnosed with maxillary posterior discrepancy
- ο Open bite class III group without maxillary posterior discrepancy (OBCIIIG-WPD) (*n* = 8): the same with the OBCIIIG-PD, but without posterior discrepancy

When both cephalometric methods (ANB and APDI) to diagnose sagittal skeletal facial growth pattern did not agree an additional evaluation that included the analysis of skeletal facial profile (sagittal relationship of the points N, A, and Pg), overjet, anteroposterior malocclusion, and soft profile convexity was considered before making a decision to which sagittal malocclusion group to assign any included case. All cephalometric radiographs were evaluated randomly.

### Maxillary posterior dentoalveolar discrepancy (MPDD)

**Fig. 1:**
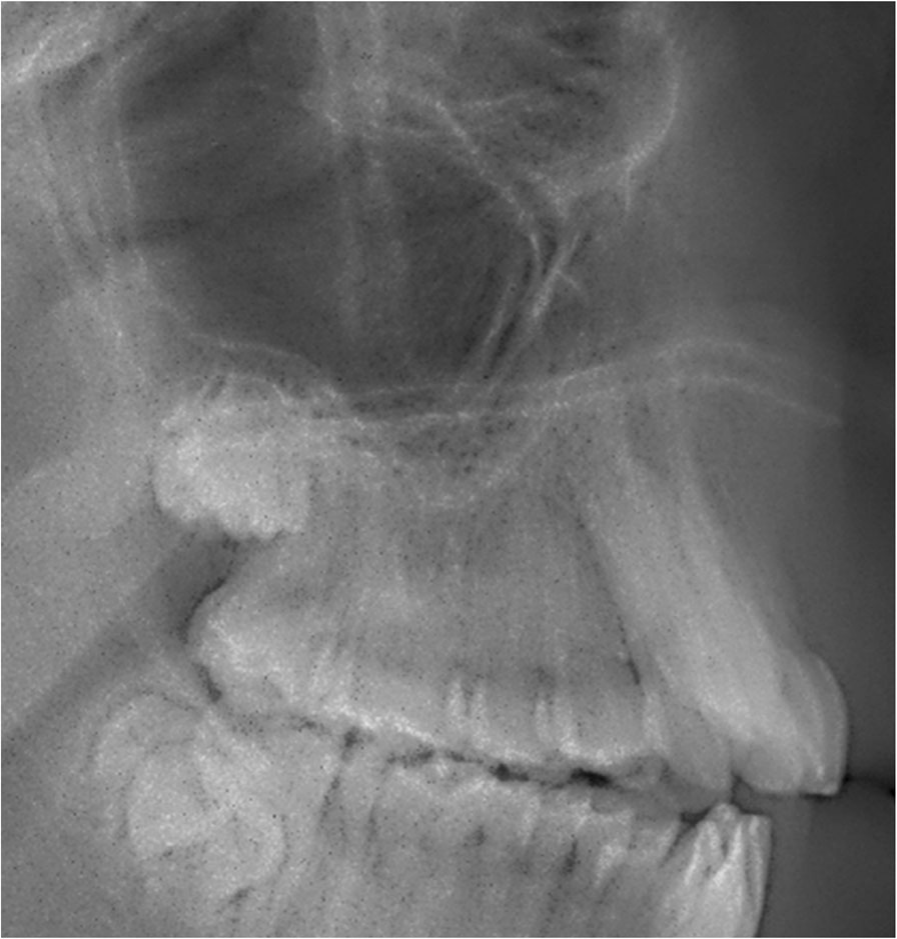
Example of MPDD. The third molar was potentially blocked by the presence of the erupted second molar

**Fig. 2:**
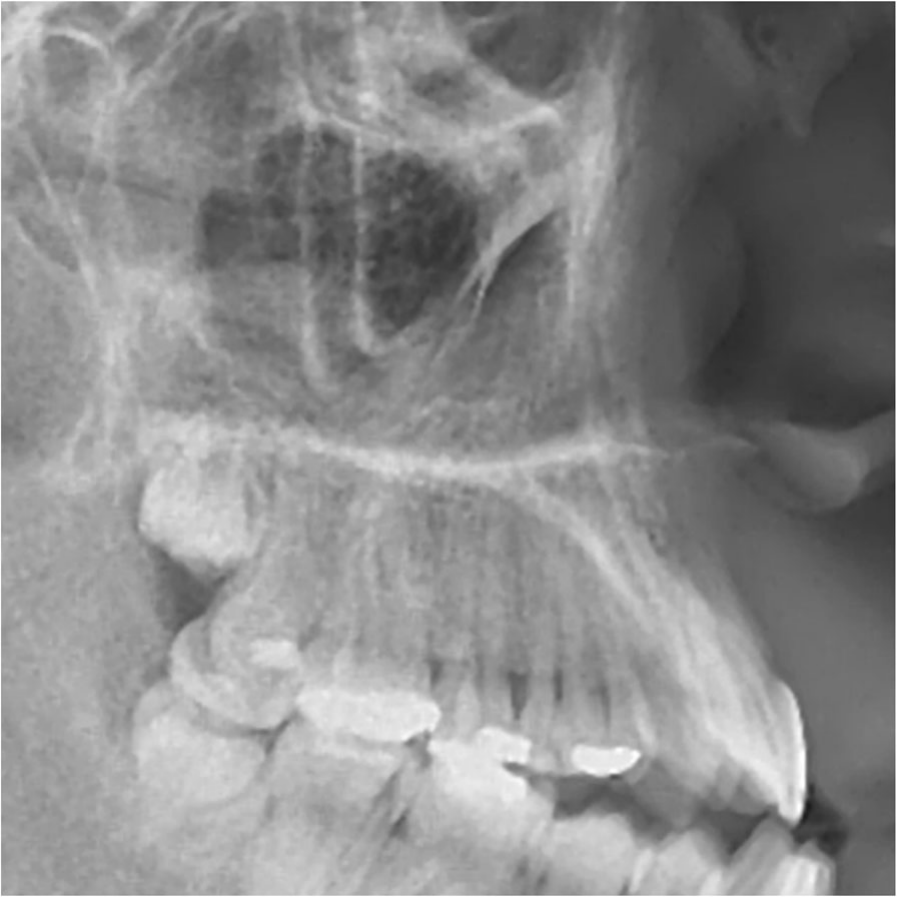
Example of MPDD. The third molar was potentially blocked by the presence of the erupted second molar

The dichotomous primary diagnosis of maxillary posterior discrepancy was made through radiographic evaluation by two calibrated examiners (LEAG, AADC). When the eruption of the maxillary third molar was apparently blocked by the presence of the erupted second, a maxillary posterior discrepancy was deemed present (Figs. 1 and 2).

**Fig. 3:**
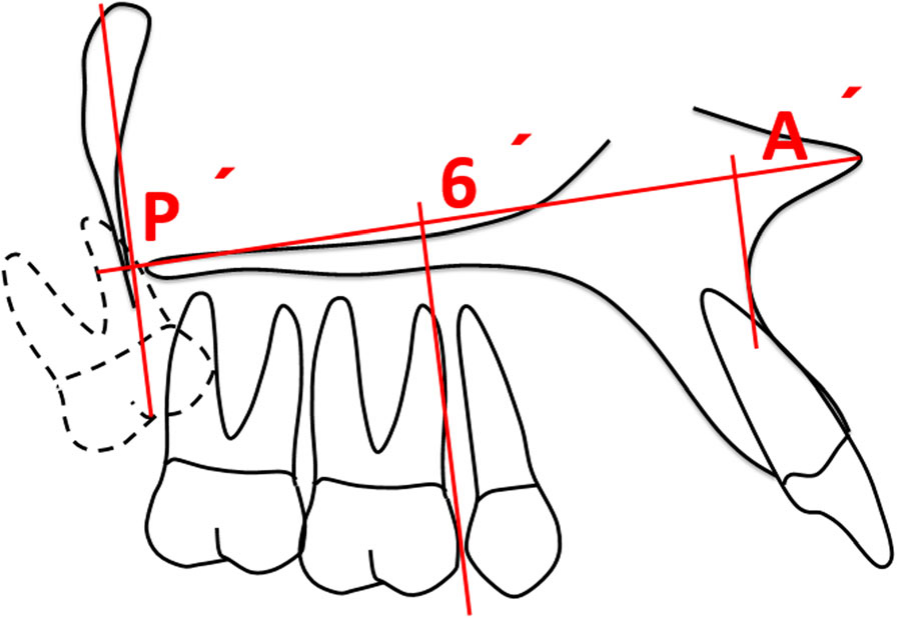
MPDD evaluated by the ratio of the anterior maxillary base length A′6′ to the maxillary base length A′P′ (A′6′/A′P′)

In addition, for statistical analysis purposes, the ratio of the anterior maxillary base length A′6′ to the maxillary base length A′P′ (A′6/ A′P′) was also calculated as a continuous variable that reflects maxillary posterior discrepancy (Fig. 3, Table 2). If the radio of the anterior maxillary base length A′6′ to the maxillary base length A′P′ (A′6/ A′P′) was greater than 0.46, then a maxillary posterior discrepancy was suggested [6, 9, 19]

### Maxillary molar sagittal angulation

**Fig. 4:**
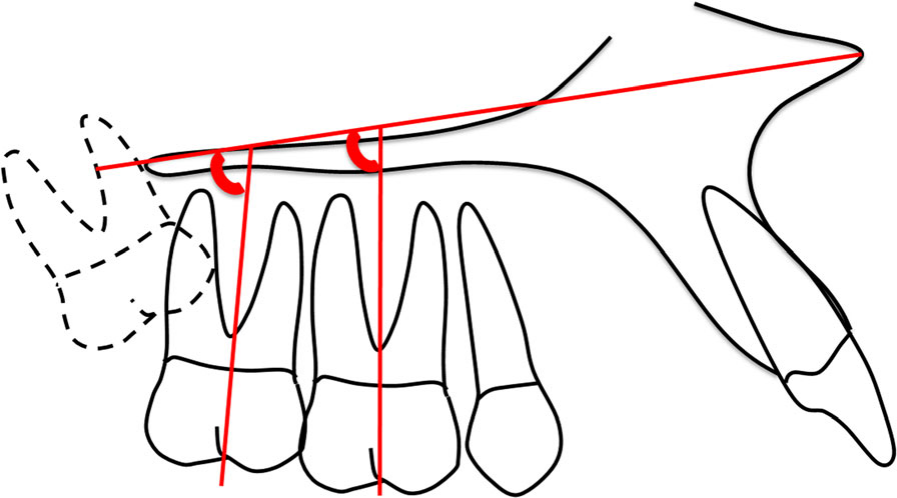
Evaluation of molar sagittal angulations

The sagittal angulations of maxillary first and second molars were measured by the angle formed by the molar axis (intercuspid groove—root bifurcation) and the palatal plane (Fig. 4).

### Statistical analysis

All statistical analyses were performed using SPSS Ver.22 for Windows (IBM SPSS, Chicago, IL, USA). Data distribution normality was according to Shapiro-Wilk tests. An independent *T* test was performed to determine differences between two groups classified by the MPDD condition (present or absent) and sagittal malocclusion. Statistical significance was set at *p* < 0.05. A principal component analysis (PCA) was used to reduce the number of variables considered during the multivariate analysis. Finally, a multivariate analysis (MANCOVA) test was applied considering the effect of SNB, ANB, APDI, A′P′, ratio (A′6′/A′P′), overbite, lower anterior facial height, ratio facial height, maxillary posterior discrepancy, and sex (reduced by the PCA from the initial cephalometric variables) on the molar sagittal angulations (outcome variable). Statistical significance was set at *p* < 0.05 for all the tests.

## Results

### Reliability

Inter and intra-examiner reliability was assessed with the intra-class correlation coefficient (ICC). All cephalometric values were greater than 0.979 (CI 95 % 0.954–0.999). In addition, the Dahlberg error was less than 1° for all angular measurements and 1 mm for all lineal measurements. All the cephalometric tracings were made with at least a 1-month interval between them and were performed by the same two different examiners.

### Outcome variables

Table 3 shows the characteristics of the six groups by skeletal facial growth pattern and maxillary posterior discrepancy.

Descriptive statistics of the outcome variables can be found in Table 4. The maxillary first and second molar angulations in individuals with maxillary posterior discrepancy had a major distal crown tipping. The maxillary first molar angulation in the OBCIG-PD was 79.74° ± 4.69° and OBCIG-WPD was 88.41° ± 4.19° (*p* < 0.001), in the OBCIIG-PD was 74.17° ± 6.15° and OBCIIG-WPD was 83.67° ± 5.55° (*p* < 0.001), and in the OBCIIIG-PD was 82.85° ± 5.50° and OBCIIIG-WPD was 89.58° ± 1.94° (*p* = 0.004). The maxillary second molar angulation in the OBCIG-PD was 71.04° ± 4.99° and OBCIG-WPD was 82.94° ± 7.75° (*p* < 0.001), in the OBCIIG-PD was 61.98 ± 7.80° and OBCIIG-WPD was 79.27° ± 7.74° (*p* < 0.001), and in the OBCIIIG-PD was 71.39° ± 7.25° and OBCIIIG-WPD was 85.59° ± 1.71° (*p* < 0.001).

**Table 4:** Maxillary molar angulation according to maxillary posterior dentoalveolar discrepancy and facial growth pattern

Measurement	Group	Mean	SD	Min	Max	*p*
Maxillary first molar angulation	OBCIG-PD	79.74	4.69	73.00	89.39	<0.001*
	OBCIG-WPD	88.41	4.19	80.92	92.85	
	OBCIIG-PD	74.17	6.15	64.42	86.95	<0.001*
	OBCIIG-WPD	83.67	5.55	72.65	92.35	
	OBCIIIG-PD	82.85	5.50	75.58	95.10	0.004*
	OBCIIIG-WPD	89.58	1.94	86.99	92.93	
Maxillary second molar angulation	OBCIG-PD	71.04	4.99	59.22	78.79	<0.001*
	OBCIG-WPD	82.94	7.75	64.24	90.06	
	OBCIIG-PD	61.98	7.80	48.45	74.49	<0.001*
	OBCIIG-WPD	79.27	7.74	62.16	94.56	
	OBCIIIG-PD	71.39	7.25	59.27	86.07	<0.001*
	OBCIIIG-WPD	85.59	1.71	83.10	88.36	

*Significant based on *T* Test

*OBCIG* open bite class I group, *OBCIIG* open bite class II group, *OBCIIIG* open bite class III group, *PD* posterior discrepancy, *WPD* without posterior discrepancy

For the final analysis, the sample was separated into two groups according to MPDD (present = 50, absent = 40), trying to know the influence of MPDD in all types of malocclusion with open bite on the angulation of the upper molars. We also found significant differences between the two groups, *p* < 0.001 (Table 5).

**Table 5:** Maxillary molar angulation according to maxillary posterior dentoalveolar discrepancy

Measurement	Group	Number	Mean	SD	Mean difference	Confidence interval 95 %		*p*
						Lower limit	Upper limit	
Maxillary first molar angulation	With maxillary posterior dentoalveolar discrepancy	50	78.43	6.46	7.61	5.07	10.13	<0.001*
	Without maxillary posterior dentoalveolar discrepancy	40	86.04	5.33				
Maxillary second molar angulation	With maxillary posterior dentoalveolar discrepancy	50	67.69	8.01	13.76	10.51	17.01	<0.001*
	Without maxillary posterior dentoalveolar discrepancy	40	81.45	7.31				

*Independent *T* test

Through a PCA (Table 6), it was determined that ANB, FMP, and ODI, as well as SNA, and A′P′, equally A′6′, and ratio (A′6′/A′P′) were significantly associated in this sample. SNB, ANB, APDI, A′P′, ratio (A′6′/A′P′), overbite, lower anterior facial height, ratio facial height maxillary posterior discrepancy, and sex were obtained after the reduction of the number of variables considered and were used in the MANCOVA. Significance was only found for maxillary posterior discrepancy (*p* < 0.001), APDI (*p* = 0.001), and ratio (A′6′/A′P′) (*p* = 0.026) for maxillary first molar angulation and APDI (*p* = 0.011) and maxillary posterior discrepancy (*p* < 0.001) for maxillary second molar angulation (Table 7).

**Table 6:** Principal component analysis to reduce the number of variables under study

Variables	Component			
	1	2	3	4
SNA	−0.11	0.79^a^	0.09	0.11
SNB	−0.80	0.47	0.24	−0.03
ANB	0.89^a^	0.15	−0.21	0.15
APDI	−0.91	0.06	0.14	−0.15
FMP	0.62^a^	−0.03	0.43	−0.37
A′P′	0.17	0.77^a^	−0.10	−0.27
A′6′	0.41	0.47	0.59^a^	0.34
Ratio	0.27	−0.07	0.70^a^	0.59
ODI	0.59^a^	0.18	−0.58	0.37
Overbite	−0.27	−0.46	0.16	0.39
Lower anterior facial height	−0.40	0.16	0.24	−0.58
Ratio facial height	−0.42	0.47	−0.32	0.44

^a^Related

**Table 7:** MANCOVA assessing maxillary first and second molar angulations based in fixed factors and co-variables

Dependent variable	Fixed factors and co-variables	*p*
Maxillary first molar angulation	Corrected model	<0.001*
	Intercept	0.025*
	SNB	0.200
	ANB	0.971
	APDI	0.001*
	A′P′	0.061
	Ratio (A′6′/A′P′)	0.026*
	Overbite	0.341
	Lower anterior facial height	0.587
	Ratio facial height	0.207
	Maxillary posterior discrepancy	<0.001*
	Sex	0.292
Maxillary second molar angulation	Corrected model	<0.001*
	Intercept	0.824
	SNB	0.613
	ANB	0.714
	APDI	0.011*
	A′P′	0.576
	Ratio (A′6′/A′P′)	0.881
	Overbite	0.229
	Lower anterior facial height	0.652
	Ratio facial height	0.238
	Maxillary posterior discrepancy	<0.001*
	Sex	0.777

*Significant based on MANCOVA test

## Discussion

The purpose of this study was to determine the effect of MPDD on the sagittal angulation of maxillary molars in skeletal open bite subjects. Overall, it was found that MPDD was associated with a significant mesial angulation of the second and first molar roots with a concurrent simultaneous distal angulation of the associated crowns. The findings are clinically relevant as they allow clinicians to consider these associations when facing patients with a potential MPDD. Their treatment decisions could be affected based on the degree of third molar angulation and how the erupted first and second molars are distally angulated in such cases. Distal molar movement in class II malocclusions should be careful in such scenario as this type of movements tends to distally angulate molar crowns. The method utilized to diagnose maxillary posterior discrepancy used a radiographic visual assessment by two calibrated examiners (LEAG, AADC), where the sagittal maxillary length and the portrayed trajectory of eruption of the third molars were considered. An unfavorable maxillary third molar eruption angulation would likely imply impaction (Figs. 1 and 2). In the literature, it has been proposed that maxillary posterior discrepancy should be determined by the ratio between the space from point A′ to mesial of the maxillary first molar, in relation to the space from point A′ to the most posterior point of the maxillary tuberosity [6, 9, 19]. When this ratio is increased, the chances of third molars having space for their eruption are diminished (Fig. 3).

The expected results, based on Kim [8] and Sato [9] hypothesis, were that there should be an increase in the mesial inclination of maxillary first and second molars (involving their crowns) that could promote posterior teeth interferences and therefore create a potential open bite scenario. However, the results of the current study suggested that maxillary molars showed more distally inclined crowns in the posterior discrepancy group. All the groups with posterior discrepancy showed significantly more distal molar crown angulation. This phenomenon supports the second hypothesis that the pressure from the erupting maxillary third molar against the anteriorly positioned molars may generate a major mesial displacement of the second and first molars roots with a concurrent simultaneous distal angulation of the associated crowns. These findings are in concordance with previous findings [1] where impacted third molars were observed to produce a distal angulation effect on the adjacent molars (involving their crowns) in subjects without open bite. Similar findings were observed in other studies [4, 23] showing that in high-angle cases, a greater degree of distal angulation of first molars was found. The latter suggested natural dentoalveolar compensation as potential explanation for the results.

### Limitations

In this study, a maxillary posterior dentoalveolar discrepancy (defined in this study as an apparent lack of space for a complete maxillary third molar eruption) could be considered equivalent to maxillary third molar impaction.

A longitudinal cohort design would generate stronger data to support or refute the evaluated hypothesis and could use CBCT if possible, trying to avoid the superimpositions, although in this paper were excluded radiographs that showed evidently this problem.

Since open bite individuals without maxillary posterior discrepancy could have unerupted third molars with available space and good eruption pattern or third molars in occlusion, ideally, a study considering the maxillary molars inclination using open bite individuals with posterior discrepancy and open bite individuals with fully erupted third molar should follow-up. Results of such studies may or not support this study’s conclusion.

Previously, two studies [1, 24] used complete root formation with the highest part of third molar below the cervical line of second molar as a criterion for third molar impaction, but they do not consider the direction of eruption that is likely directly related to the impaction potential. In addition, the third molar’s roots are not fully formed until 20 to 22 years of age [24]. Accordingly, third molar impaction could be over diagnosed when examining subjects younger than 20 years old. In the current study, the subjects were between 15 to 30 years old. This could be considered a weakness, but it was, at least partially, controlled considering the eruption trajectory of third molar crowns as an independent factor from complete root formation.

The association between the severity of maxillary molar crown distal inclination and the degree of MPDD was not evaluated in this study.

## Conclusions

The maxillary posterior dentoalveolar discrepancy generates a major mesial displacement of the second and first molar roots with a concurrent simultaneous distal angulation of the associated crowns in individuals with skeletal open bite.

## Abbreviations

MPDD: Maxillary posterior dentoalveolar discrepancy
PD: Posterior discrepancy
